# Vague Entropy Measure for Complex Vague Soft Sets

**DOI:** 10.3390/e20060403

**Published:** 2018-05-24

**Authors:** Ganeshsree Selvachandran, Harish Garg, Shio Gai Quek

**Affiliations:** 1 Department of Actuarial Science and Applied Statistics, Faculty of Business & Information Science, UCSI University, Jalan Menara Gading, Cheras 56000, Kuala Lumpur, Malaysia; 2 School of Mathematics, Thapar Institute of Engineering & Technology (Deemed University), Patiala 147004, Punjab, India; 3 A-Level Academy, UCSI College KL Campus, Lot 12734, Jalan Choo Lip Kung, Taman Taynton View, Cheras 56000, Kuala Lumpur, Malaysia

**Keywords:** vague entropy, distance induced vague entropy, distance, complex fuzzy set, complex vague soft set

## Abstract

The complex vague soft set (CVSS) model is a hybrid of complex fuzzy sets and soft sets that have the ability to accurately represent and model two-dimensional information for real-life phenomena that are periodic in nature. In the existing studies of fuzzy and its extensions, the uncertainties which are present in the data are handled with the help of membership degree which is the subset of real numbers. However, in the present work, this condition has been relaxed with the degrees whose ranges are a subset of the complex subset with unit disc and hence handle the information in a better way. Under this environment, we developed some entropy measures of the CVSS model induced by the axiomatic definition of distance measure. Some desirable relations between them are also investigated. A numerical example related to detection of an image by the robot is given to illustrate the proposed entropy measure.

## 1. Introduction

Classical information measures deal with information which is precise in nature, while information theory is one of the trusted ways to measure the degree of uncertainty in data. In our day-to-day life, uncertainty plays a dominant role in any decision-making process. In other words, due to an increase of the system day-by-day, decision makers may have to give their judgments in an imprecise, vague and uncertain environment. To deal with such information, Zadeh [1] introduced the theory of fuzzy sets (FSs) for handling the uncertainties in the data by defining a membership function with values between 0 and 1. In this environment, Deluca and Termini [2] proposed a set of axioms for fuzzy entropy. Liu [3] and Fan and Xie [4] both studied information measures related to entropy, distance, and similarity for fuzzy sets. With the growing complexities, researchers are engaged in extensions such as intuitionistic fuzzy set (IFS) [5], vague set (VS) [6], interval-valued IFS [7] to deal with the uncertainties. Under these extensions, Szmidt and Kacprzyk [8] extended the axioms of Deluca and Termini [2] to the IFS environment. Later on, corresponding to Deluca and Termini’s [2] fuzzy entropy measure, Vlachos and Sergiadis [9] extended their measure in the IFS environment. Burillo and Bustince [10] introduced the entropy of intuitionistic fuzzy sets (IFSs), as a tool to measure the degree of intuitionism associated with an IFS. Garg et al. [11] presented a generalized intuitionistic fuzzy entropy measure of order *α* and degree *β* to solve decision-making problems. In addition to the mentioned examples, other authors have also addressed the problem of decision-making by using the different information measures [12,13,14,15,16,17,18,19,20,21,22,23,24,25].

All the above-defined work is successfully applied to the various disciplines without considering the parameterization factor during the analysis. Therefore, under some certain cases, these existing theories may be unable to classify the object. To cope with such situations, many researchers are paying more attention to soft set (SS) theory [26]. After its discovery, researchers are engaged in its extensions. For instance, Maji et al. [27,28] combined the theory of SSs with FSs and IFSs and came up with a new concept of the fuzzy soft set (FSS) and intuitionistic fuzzy soft set (IFSS). Further, the concept of the hybridization of the SSs with the others, such as generalized fuzzy soft set [29,30], generalized intuitionistic fuzzy soft set [31,32], distance measures [33,34,35,36], and fuzzy number intuitionistic fuzzy soft sets [37] plays a dominant role during the decision making process. IFSS plays a dominant role in handling the uncertainties in the data by incorporating the idea of the expert as well as the parametric factors. In that environment, Arora and Garg [38,39] presented some aggregation operators for intuitionistic fuzzy soft numbers. Garg and Arora [40] presented some non-linear methodology for solving decision-making problems in an IFSS environment. In terms of the information measures, Garg and Arora [34] developed various distance and similarity measures for dual hesitant FSS. Recently, Garg and Arora [41] presented Bonferroni mean aggregation operators for an IFSS environment. Apart from these, vague soft set [42] is an alternative theory which is the hybridization of the vague set [6] and soft set [26]. In this field, Chen [43] developed some similarity measures for vague sets. Wang and Qu [44] developed some entropy, similarity and distance measures for vague sets. Selvachandran et al. [45] introduced distance induced entropy measures for generalized intuitionistic fuzzy soft sets.

The above theories using FSs, IFSs, IFSSs, VSs, FSSs are widely employed by researchers but they are able to handle only the uncertainty in the data. On the other hand, none of these models will be able to handle the fluctuations of the data at a given phase of time during their execution, but in today’s life, the uncertainty and vagueness of the data changes periodicity with the passage of time and hence the existing theories are unable to consider this information. To overcome this deficiency, Ramot et al. [46] presented a complex fuzzy set (CFS) in which the range of membership function is extended from real numbers to complex numbers with the unit disc. Ramot et al. [47] generalized traditional fuzzy logic to complex fuzzy logic in which the sets used in the reasoning process are complex fuzzy sets, characterized by complex valued membership functions. Later on, Greenfield et al. [48] extended the concept of CFS by taking the grade of the membership function as an interval-number rather than single numbers. Yazdanbakhsh and Dick [49] conducted a systematic review of CFSs and logic and discussed their applications. Later on, Alkouri and Salleh [50] extended the concepts of CFS to complex intuitionistic fuzzy (CIF) sets (CIFSs) by adding the degree of complex non-membership functions and studied their basic operations. Alkouri and Salleh [51] introduced the concepts of CIF relation, composition, projections and proposed a distance measure between the two CIFSs. Rani and Garg [52] presented some series of distance measures for a CIFS environment. Kumar and Bajaj [53] proposed some distance and entropy measures for CIF soft sets. In these theories, a two-dimensional information (amplitude and phase terms) are represented as a single set. The function of the phase term is to model the periodicity and/seasonality of the elements. For instance, when dealing with an economics-related situation, the phase term represents the time taken for the change in an economic variable to impact the economy. On the other hand, in robotics, the phase term can represent direction, whereas in image processing, the phase term can represent the non-physical attributes of the image.

As an alternative to these theories, the concept of the complex vague soft set (CVSS) [54] handles the two-dimensional information by combining the properties of CFSs [46], soft sets [26] and vague sets [6]. The CVSSs differs from the existing sets with the features that they contain: (1) an interval-based membership structure that provides users with the means of recording their hesitancy in the process of assigning membership values for the elements; (2) the ability to handle the partial ignorance of the data; (3) adequate parameterization abilities that allow for a more comprehensive representation of the parameters. Selvachandran et al. [54,55] investigated complex vague soft sets (CVSSs). Selvachandran et al. [56] presented similarity measures for CVSSs and their applications to pattern recognition problems.

Thus, motivated from the concept of CVSS, the focus of this work is to explore the structural characteristics of CVSSs and to present some information measures for handling the uncertainties in the data. Per our knowledge, in the aforementioned studies, the information measures cannot be utilized to handle the CVSS information. Thus, in order to achieve this, we develop the axiomatic definition of the distance and entropy measures between CVSSs and hence propose some new entropy measures. Some of the algebraic properties of these measures and the relations between them are also verified. The proposed measures have the following characteristics: (1) they serve as a complement to the CVSS model and its relations in representing and modeling time-periodic phenomena; (2) they have elegant properties that increase their reach and applicability; (3) they have important applications in many real-world problems in the areas of image detection, pattern recognition, image processing; (4) they add to the existing collection of methodologies and techniques in artificial intelligence and soft computing, where it is often necessary to determine the degree of vagueness of the data, in order to make optimal decisions. This provides support of the increasingly widespread trend in the use of mathematical tools to complement scientific theories and existing procedures, in the handling and solving of real-life problems that involve vague, unreliable and uncertain two-dimensional information. Furthermore, an effort has been put forth to solve the classification problem in multi-dimensional complex data sets. To elaborate the proposed method, we will be focusing on the representation and recognition of digital images defined by multi-dimensional complex data sets using the properties of CVSSs and a new distance and entropy measure for this model.

The rest of the manuscript is organized as follows: in Section 2, we briefly review the basic concepts of SSs and CVSSs. In Section 3, we define the axiomatic definition of the distance and entropy measures for CVSSs. In Section 4, some basic relationships between the distance and entropy measures are defined. In Section 5, the utility of the CVSS model and its entropy measure is illustrated by applying it in a classification of the digital image with multi-dimensional data. Finally, conclusions and future work are stated in Section 6.

## 2. Preliminaries

In this section, we briefly reviewed some basic concepts related to the VSs, SSs, CVSSs defined over the universal set *U*.

**Definition 1** **[6].** *A vague set (VS) V in U is characterized by the truth and falsity membership functions t_V_, f_V_: U*→[0.1] *with t_V_*(*x*) + *f_V_*(*x*) ≤ 1 *for any x* ∈ *U*. *The values assigned corresponding to t_V_*(*x*) *and f_V_*(*x*) *are the real numbers of* [0, 1]. *The grade of membership for x can be located in* [*t_V_*(*x*), 1 − *f_V_*(*x*)] *and the uncertainty of x is defined as* (1 − *f_V_*(*x*)) − *t_V_*(*x*).

It is clearly seen from the definition that VSs are the generalization of the fuzzy sets. If we assign 1 − *f_V_*(*x*) to be 1 − *t_V_*(*x*) then VS reduces to FS. However, if we set 1 − *t_V_*(*x*) to be *ν_A_*(*x*) (called the non-membership degree) then VS reduces to IFS. On the other hand, if we set $t_{V}\left(x\right)=\mu_{V}^{L}\left(x\right)$ and $1-f_{V}\left(x\right)=\mu_{V}^{U}\left(x\right)$ then VS reduces to interval-valued FS. Thus, we conclude that VSs are the generalization of the FSs, IFSs and interval-valued FSs.

**Definition 2** **[6].** *Let*$A=\{<x,[t_{A}(x),1-f_{A}(x)]>:x\in U\}$*and*$B=\{<x,[t_{B}(x),1-f_{B}(x)]>:x\in U\}$*be two VSs defined on*U*then the basic operational laws between them are defined as follows*:

*(i)*  *A* ⊆ *B if* *t_A_*(*x*) ≤ *t_B_*(*x*) *and* 1 − *f_A_*(*x*) ≤ 1 − *f_B_*(*x*) *for all x*.
*(ii)*  *Complement: A^C^* = {<*x*, [*f_A_*(*x*), 1 − *t_A_*(*x*)]>: *x* ∈ *U*}.
*(iii)*  *Union: A*∪*B* = {<*x*, [max(*t_A_*(*x*), *t_B_*(*x*)),max(1 − *f_A_*(*x*),1 − *f_B_*(*x*))]>: *x* ∈ *U*}
*(iv)*  *Intersection: A*∩*B* = {<*x*, [min(*t_A_*(*x*), *t_B_*(*x*)), min(1 − *f_A_*(*x*),1 − *f_B_*(*x*))]>: *x* ∈ *U*}

**Definition 3** **[26].** *Let*P(U)*denote the power set of*U. *A pair*(F,A)*is called a soft set (SS) over*V*where*F*is a mapping given by*F:A→P(U).

**Definition 4** **[42].** *Let*V(U)*be the power set of VSs over*U. *A pair*$\left(\hat{F},A\right)$*is called a vague soft set (VSS) over*U,*where*$\hat{F}$*is a mapping given by*$\hat{F}:A\to V\left(U\right).$*Mathematically, VSS can be defined as follows*:

$$\left(\hat{F},\;A\right)=\left\{\langle x,\;\left[t_{{\hat{F}}_{\left(e\right)}}\left(x\right),\;1-f_{{\hat{F}}_{\left(e\right)}}\left(x\right)\right]\rangle :x\in U,\;e\in A\right\}$$

*It is clearly seen that this set is the hybridization of the SSs and VSs*.

**Definition 5** **[57].** *A complex vague set (CVS) is defined as an ordered pair defined as*

$$A=\left\{\langle x,\left[t_{A}\left(x\right),\;1-f_{A}\left(x\right)\right]\rangle :x\in U\right\}$$

*where* $t_{A}:U\to \left\{a:a\in C,\left|a\right|\leq 1\right\},\;f_{A}:U\to \left\{a:a\in X,\;\left|a\right|\leq 1\right\}$ *are the truth and falsity membership functions with unit disc and are defined as* $t_{A}\left(x\right)=r_{t_{A}}\left(x\right).e^{iw_{t_{A}}^{r}\left(x\right)}$ *and* $1-f_{A}\left(x\right)=\left(1-k_{f_{A}}\left(x\right)\right).e^{i\left(2\pi -w_{f_{A}}^{k}\left(x\right)\right)}$ *where* $i=\sqrt{-1}.$

**Definition 6** **[54].** *Let*P(U)*denote the complex vague power set of*U*and*E*be the set of parameters. For any*A⊂E, a*pair*(F, A)*is called a complex vague soft set (CVSS) over*U,*where*F:A→P(U), *defined as*:

$$F\left(x_{j}\right)=\left\{\left(x_{j},\;\left[r_{t_{F_{a}}}\left(x_{j}\right),\;1-k_{t_{F_{a}}}\left(x_{j}\right)\right].\;e^{i\;\left[w_{t_{F_{a}}}^{r}\left(x_{j}\right),\;2\pi -w_{f_{F_{a}}}^{k}\left(x_{j}\right)\right]}\right)\;:x_{j}\in U\right\}$$

*where*j=1, 2, 3, …*is the number of parameters,*$\left[r_{t_{F_{a}}}\left(x\right),\;1-k_{t_{F_{a}}}\left(x\right)\right]$*are real-valued*∈[0, 1], *the phase terms*$\left[w^{r}{}_{t_{F_{a}}}\left(x\right),\;2\pi -w^{k}{}_{f_{F_{a}}}\left(x\right)\right]$*are real-valued in the interval*(0, 2π],$0\leq r_{t_{F_{a}}}\left(x\right)+k_{F_{a}}\left(x\right)\leq 1$*and*$i=\sqrt{-1}.$

The major advantages of the CVSS are that it represents two-dimensional information in a single set and each object is characterized in terms of its magnitude as well as its phase term. Further, the soft set component in CVSS provides an adequate parameterization tool to represent the information.

**Definition 7** **[54].** *Let two CVSSs* (F, A) *and* (G, B) *over* U, *the basic operations between them are defined as*

*(i)*  (F, A)⊂(G,B) *if and only if the following conditions are satisfied for all* x ∈U:
  *(a)*  $r_{t_{F_{a}}}\left(x\right)\leq r_{t_{G_{b}}}\left(x\right)$*and*$k_{f_{G_{b}}}\left(x\right)\leq k_{f_{F_{a}}}\left(x\right)$;
  *(b)*  $w_{t_{F_{a}}}^{r}\left(x\right)\leq w_{t_{G_{b}}}^{r}\left(x\right)$*and*$w_{f_{G_{b}}}^{k}\left(x\right)\leq w_{f_{F_{a}}}^{k}\left(x\right)$.
*(ii)*  *Null CVSS:*(F, A)=ϕ*if*$r_{t_{F_{a}}}\left(x\right)=0,$$k_{f_{F_{a}}}\left(x\right)=1$*and*$w_{t_{F_{a}}}^{r}\left(x\right)=0\pi ,\;w_{f_{F_{a}}}^{k}\left(x\right)=2\pi$*for all*x∈U.
*(iii)*  *Absolute CVSS:* (F, A)=1 *if* $r_{t_{F_{a}}}\left(x\right)=1,$ $k_{f_{F_{a}}}\left(x\right)=0$ *and* $w_{t_{F_{a}}}^{r}\left(x\right)=2\pi ,$ $w_{f_{F_{a}}}^{k}\left(x\right)=0\pi$ *for all* x∈U.

## 3. Axiomatic Definition of Distance Measure and Vague Entropy

Let *E* be a set of parameters and *U* be the universe of discourse. In this section, we present some information measures namely distance and entropy for the collections of CVSSs, which are denoted by CVSS(*U*).

**Definition** **8.** *Let*(F, A), (G, B), (H, C) ∈CVSS(U). *A complex-value function*d :CVSS(U) ×CVSS(U) →{a, a∈U, |a|≤1}*is called a distance measure between CVSSs if it satisfies the following axioms*:

*(D1)*  d((F, A), (G, B))=d((G, B), (F, A))
*(D2)*  d((F, A), (G, B))=0⟺(F, A)=(G, B)
*(D3)*  d((F, A), (G, B))=1⟺∀ e∈E, x∈U,*both*(F, A)*and*(G, B)*are crisp sets in*U, i.e.,
  $\left(F,\;A\right)=\left\{\left(x,\;\left[0,\;0\right]e^{i\left[0\pi ,\;0\pi \right]}\right)\right\}$*and*$\left(G,\;B\right)=\left\{\left(x,\;\left[1,\;1\right]e^{i\left[2\pi ,\;2\pi \right]}\right)\right\}$,
  *or*$\left(F,\;A\right)=\left\{\left(x,\;\left[0,\;0\right]e^{i\left[2\pi ,\;2\pi \right]}\right)\right\}$*and*$\left(G,\;B\right)=\left\{\left(x,\;\left[1,\;1\right]e^{i\left[0\pi ,\;0\pi \right]}\right)\right\}$,
  *or*$\left(F,\;A\right)=\left\{\left(x,\;\left[1,\;1\right]e^{i\left[2\pi ,\;2\pi \right]}\right)\right\}$*and*$\left(G,\;B\right)=\left\{\left(x,\;\left[0,\;0\right]e^{i\left[0\pi ,\;0\pi \right]}\right)\right\}$,
  *or*$\left(F,\;A\right)=\left\{\left(x,\;\left[1,\;1\right]e^{i\left[0\pi ,\;0\pi \right]}\right)\right\}$*and*$\left(G,\;B\right)=\left\{\left(x,\;\left[0,\;0\right]e^{i\left[2\pi ,\;2\pi \right]}\right)\right\}.$
*(D4)*  *If*(F, A)⊆(G, B)⊆(H, C),*then*d((F, A), (H, C))≥max(d((F, A), (G, B)), d((G, B), (H, C))).

Next, we define the axiomatic definition for the vague entropy for a CVSS.

**Definition** **9.** *A complex-valued function* M:CVSS(U)→{a:a∈ℂ, |a|≤1} *is called vague entropy of* *CVSSs, if it satisfies the following axioms for any* (F, A), (G, B)∈CVSS(U).

*(M1)*  0≤|M(F, A)|≤1.
*(M2)*  M(F, A)=0 ⟺(F, A) *is a crisp set on* U *for all* a∈A *and* x∈U, *i.e.,* $r_{t_{F_{a}}}\left(x\right)=1,\;k_{f_{F_{a}}}\left(x\right)=0$ *and* $w_{t_{F_{a}}}^{r}\left(x\right)=2\pi ,\;w_{f_{F_{a}}}^{k}\left(x\right)=0\pi$ *or* $r_{t_{F_{a}}}\left(x\right)=1,\;k_{f_{F_{a}}}\left(x\right)=0$ *and* $w_{t_{F_{a}}}^{r}\left(x\right)=0\pi ,\;w_{f_{F_{a}}}^{k}\left(x\right)=2\pi$ *or* $r_{t_{F_{a}}}\left(x\right)=0,\;k_{f_{F_{a}}}\left(x\right)=1$ *and* $w_{t_{F_{a}}}^{r}\left(x\right)=0\pi ,\;w_{f_{F_{a}}}^{k}\left(x\right)=2\pi$ *or* $r_{t_{F_{a}}}\left(x\right)=0,\;k_{f_{F_{a}}}\left(x\right)=1$ *and* $w_{t_{F_{a}}}^{r}\left(x\right)=2\pi ,\;w_{f_{F_{a}}}^{k}\left(x\right)=0\pi .$
*(M3)*  M(F, A)=1⟺∀ a ∈A*and*x∈U,(F, A) is completely vague
*i.e.,* $r_{t_{F_{a}}}\left(x\right)=k_{f_{F_{a}}}\left(x\right)$ *and* $w_{t_{F_{a}}}^{r}\left(x\right)=w_{f_{F_{a}}}^{k}\left(x\right).$
*(M4)*  $M\left(F,\;A\right)=M\left({\left(F,\;A\right)}^{c}\right)$
*(M5)*  *If the following two cases holds for all* a∈A *and* x∈U,
  $$Case\;1:r_{t_{F_{a}}}\left(x\right)\leq r_{t_{G_{b}}}\left(x\right),\;k_{f_{F_{a}}}\left(x\right)\geq k_{f_{G_{b}}}\left(x\right)whenever\;r_{t_{G_{b}}}\left(x\right)\leq k_{f_{G_{b}}}\left(x\right);$$
  $$and\;w_{t_{F_{a}}}^{r}\left(x\right)\leq w_{t_{G_{b}}}^{r}\left(x\right),\;w_{f_{F_{a}}}^{k}\left(x\right)\geq w_{f_{G_{b}}}^{k}\left(x\right)\;whenever\;w_{t_{G_{b}}}^{r}\left(x\right)\leq w_{f_{G_{b}}}^{k}\left(x\right);$$
  $$Case\;2:r_{t_{F_{a}}}\left(x\right)\geq r_{t_{G_{b}}}\left(x\right),\;k_{f_{F_{a}}}\left(x\right)\leq k_{f_{G_{b}}}\left(x\right)whenever\;r_{t_{G_{b}}}\left(x\right)\geq k_{f_{G_{b}}}\left(x\right)$$
  $$and\;w_{t_{F_{a}}}^{r}\left(x\right)\geq w_{t_{G_{b}}}^{r}\left(x\right),w_{f_{F_{a}}}^{k}\left(x\right)\leq w_{f_{G_{b}}}^{k}\left(x\right)whenever\;w_{t_{G_{b}}}^{r}\left(x\right)\geq w_{f_{G_{b}}}^{k}\left(x\right);$$

*then* M(F, A)≤M(G, B).

Based on this definition, it is clear that a value close to 0 indicates that the CVSS has a very low degree of vagueness whereas a value close to the 1 implies that the CVSS is highly vague. For all x∈U, the nearer $r_{t_{F_{a}}}\left(x\right)$ is to $k_{f_{F_{a}}}\left(x\right),$ the larger the vague entropy measure and it reaches a maximum when $r_{t_{F_{a}}}\left(x\right)=k_{f_{F_{a}}}\left(x\right).$ Condition M5 on the other hand, is slightly different as it is constructed using the sharpened version of a vague soft set as explained in Hu et al. [58], instead of the usual condition of (F, A)⊂(G, B) implies that the entropy of (F, A) is higher than the entropy of (G, B). In [58], Hu et al. proved that this condition is inaccurate and provided several counter-examples to disprove this condition. Subsequently, they replaced this flawed condition with two new cases. We generalized these two cases to derive condition (M5) in this paper, in a bid to increase the accuracy of our proposed vague entropy. We refer the readers to [58] for further information on these revised conditions. 

## 4. Relations between the Proposed Distance Measure and Vague Entropy

In the following, let U be universal and ϕ be empty over CVSSs. Then based on the above definition, we define some of the relationship between them as follows:

**Theorem** **1.** *Let*(F, A)*be CVSS and*d*is the distance measure between CVSSs, then the equations*$M_{1},\;M_{2}$*and*$M_{3}$ defined as below

(i) $M_{1}\left(F,\;A\right)=1-d\left(\left(F,\;A\right),\;{\left(F,\;A\right)}^{c}\right)$
(ii) $M_{2}\left(F,\;A\right)=d\left(\left(F,\;A\right)\cup^{\text{​}}{\left(F,\;A\right)}^{c},\;U\right)$
(iii) $M_{3}\left(F,\;A\right)=1-d\left(\left(F,\;A\right)\cup^{\text{​}}{\left(F,\;A\right)}^{c},\;\left(F,\;A\right)\cap^{\text{​}}{\left(F,\;A\right)}^{c}\right)$

*are the valid vague entropies of CVSSs*.

**Proof.**  Here, we shall prove only the part (i), while others can be proved similarly.

It is clearly seen from the definition of vague entropies that $M_{1}$ satisfies conditions (M1) to (M4). So we need to prove only (M5). For it, consider the two cases stated in Definition 9. We only prove that the condition (M5) is satisfied for Case 1; the proof for Case 2 is similar and is thus omitted.

From the conditions given in Case 1 of (M5), we obtain the following relationship:

$$r_{t_{F_{a}}}\left(x\right)\leq r_{t_{G_{b}}}\left(x\right)\leq k_{f_{G_{b}}}\left(x\right)\leq k_{f_{F_{a}}}\left(x\right)$$

$$\mathrm{and}\;w_{t_{F_{a}}}^{r}\left(x\right)\leq w_{t_{G_{b}}}^{r}\left(x\right)\leq w_{f_{G_{b}}}^{k}\left(x\right)\leq w_{f_{F_{a}}}^{k}\left(x\right).$$

Therefore, we have:

$$\phi \subset \left(F,\;A\right)\subset \left(G,\;B\right)\subset {\left(G,\;B\right)}^{c}\subset {\left(F,\;A\right)}^{c}\subset U.$$

Hence, it follows that:

$$\left(\left(F,\;A\right),\;{\left(F,\;A\right)}^{c}\right)\geq d\left(\left(G,\;B\right),\;{\left(G,\;B\right)}^{c}\right).$$

Now, by definition of $M_{1},$ we have:

$$M_{1}\left(F,\;A\right)=1-d\left(\left(F,\;A\right),\;{\left(F,\;A\right)}^{c}\right)$$

$$\leq 1-d\left(\left(G,\;B\right),\;{\left(G,\;B\right)}^{c}\right)$$

$$=M_{1}\left(G,\;B\right).$$

This completes the proof.  ☐

**Theorem** **2.** *If d is the distance measure between CVSSs, then*:

$$M_{4}\left(F,\;A\right)=\frac{d\left(\left(F,\;A\right)\cup^{\text{​}}{\left(F,\;A\right)}^{c},\;U\right)}{d\left(\left(F,\;A\right)\cap^{\text{​}}{\left(F,\;A\right)}^{c},\;U\right)}$$

*is a vague entropy of CVSSs*.

**Proof.**  For two CVSSs (F,A) & (G,B), clearly seen that $M_{4}$ satisfies conditions (M1)–(M4). So, it is enough to prove that $M_{4}$ satisfy the condition (M5).

Consider the case:

$$r_{t_{F_{a}}}\left(x\right)\leq r_{t_{G_{b}}}\left(x\right),\;k_{f_{F_{a}}}\left(x\right)\geq k_{f_{G_{b}}}\left(x\right)\;\mathrm{whenever}\;r_{t_{G_{b}}}\left(x\right)\leq k_{f_{G_{b}}}\left(x\right)$$

$$\mathrm{and}\;w_{t_{F_{a}}}^{r}\left(x\right)\leq w_{t_{G_{b}}}^{r}\left(x\right),\;w_{f_{F_{a}}}^{k}\left(x\right)\geq w_{f_{G_{b}}}^{k}\left(x\right)\;\;\mathrm{whenever}\;w_{t_{G_{b}}}^{r}\left(x\right)\leq w_{f_{G_{b}}}^{k}\left(x\right)$$

which implies that:

$$r_{t_{F_{a}}}\left(x\right)\leq r_{t_{G_{b}}}\left(x\right)\leq k_{f_{G_{b}}}\left(x\right)\leq k_{f_{F_{a}}}\left(x\right)$$

$$\mathrm{and}\;w_{t_{F_{a}}}^{r}\left(x\right)\leq w_{t_{G_{b}}}^{r}\left(x\right)\leq w_{f_{G_{b}}}^{k}\left(x\right)\leq w_{f_{F_{a}}}^{k}\left(x\right).$$

Thus, we obtain:

$$\phi \subset \left(F,\;A\right)\cap^{\text{​}}{\left(F,\;A\right)}^{c}\subset \left(G,\;B\right)\cap^{\text{​}}{\left(G,\;B\right)}^{c}\subset \left(G,\;B\right)\cup^{\text{​}}{\left(G,\;B\right)}^{c}\subset \left(F,\;A\right)\cup^{\text{​}}{\left(F,\;A\right)}^{c}\subset U.$$

Therefore, we have:

$$d\left(\left(F,\;A\right)\cup^{\text{​}}{\left(F,\;A\right)}^{c},\;U\right)\leq d\left(\left(G,\;B\right)\cup^{\text{​}}{\left(G,\;B\right)}^{c}\;U\right)$$

$$\mathrm{and}\;d\left(\left(F,\;A\right)\cap^{\text{​}}{\left(F,\;A\right)}^{c},\;U\right)\leq d\left(\left(G,\;B\right)\cap^{\text{​}}{\left(G,\;B\right)}^{c}\;U\right).$$

Hence, by definition of $M_{4}$, we have:

$$M_{4}\left(F,\;A\right)=\frac{d\left(\left(F,\;A\right)\cup^{\text{​}}{\left(F,\;A\right)}^{c},\;U\right)}{d\left(\left(F,\;A\right)\cap^{\text{​}}{\left(F,\;A\right)}^{c},\;U\right)}$$

$$\leq \frac{d\left(\left(G,\;B\right)\cup^{\text{​}}{\left(G,\;B\right)}^{c}\;U\right)}{d\left(\left(G,\;B\right)\cap^{\text{​}}{\left(G,\;B\right)}^{c}\;U\right)}$$

$$=M_{4}\left(G,\;B\right)$$

Similarly, we can obtain for other case i.e., when $r_{t_{F_{a}}}\left(x\right)\geq r_{t_{G_{b}}}\left(x\right),\;k_{f_{F_{a}}}\left(x\right)\leq k_{f_{G_{b}}}\left(x\right)$ whenever $r_{t_{G_{b}}}\left(x\right)\geq k_{f_{G_{b}}}\left(x\right)$ and $w_{t_{F_{a}}}^{r}\left(x\right)\geq w_{t_{G_{b}}}^{r}\left(x\right),$ $w_{f_{F_{a}}}^{k}\left(x\right)\leq w_{f_{G_{b}}}^{k}\left(x\right)$ whenever $w_{t_{G_{b}}}^{r}\left(x\right)\geq w_{f_{G_{b}}}^{k}\left(x\right)$, we have $M_{4}\left(F,A\right)\leq M_{4}\left(G,\;B\right)$. Hence (M5) satisfied.

Therefore, $M_{4\;}$ is a valid entropy measure.  ☐

**Theorem** **3.** *For CVSS*(F, A)*and if*d*is the distance measure between CVSSs, then*:

$$M_{5}\left(F,\;A\right)=\frac{d\left(\left(F,\;A\right)\cap^{\text{​}}{\left(F,\;A\right)}^{c},\;\phi \right)}{d\left(\left(F,\;A\right)\cup^{\text{​}}{\left(F,\;A\right)}^{c},\;\phi \right)}$$

*is a vague entropy of CVSSs*.

**Proof.**  It can be obtained as similar to Theorem 2, so we omit here.  ☐

**Theorem** **4.** *For two CVSSs*(F,A)*and*(G,B). *If*d*is a distance measure between CVSSs such that*:

$$d\left(\left(F,\;A\right),\;\left(G,\;B\right)\right)=d\left({\left(F,\;A\right)}^{c},\;{\left(G,\;B\right)}^{c}\right),$$

*then the entropies*$M_{4}$*and*$M_{5}$*satisfies the equation*$M_{4}=M_{5}.$

**Proof.**  By definition of $M_{4}$ and $M_{5}$, we have:

$$M_{4}\left(F,\;A\right)=\frac{d\left(\left(F,\;A\right)\cup^{\text{​}}{\left(F,\;A\right)}^{c},\;U\right)}{d\left(\left(F,\;A\right)\cap^{\text{​}}{\left(F,\;A\right)}^{c},\;U\right)}$$

$$=\frac{d\left({\left(\left(F,\;A\right)\cup^{\text{​}}{\left(F,\;A\right)}^{c}\right)}^{c},\;U^{c}\right)}{d\left({\left(\left(F,\;A\right)\cap^{\text{​}}{\left(F,\;A\right)}^{c}\right)}^{c},\;U^{c}\right)}$$

$$=\frac{d\left(\left(F,\;A\right)\cap^{\text{​}}{\left(F,\;A\right)}^{c},\;\phi \right)}{d\left(\left(F,\;A\right)\cup^{\text{​}}{\left(F,\;A\right)}^{c},\;\phi \right)}$$

$$=M_{5}\left(F,\;A\right).\;☐$$

**Theorem** **5.** *For a CVSS*(F,A),*if*d*is the distance measure between CVSSs and satisfies*:

d((F, A), U)=d((F, A), ϕ),

*Then*:

$$M_{6}\left(F,\;A\right)=\frac{d\left(\left(F,\;A\right)\cup^{\text{​}}{\left(F,\;A\right)}^{c},\;U\right)}{d\left(\left(F,\;A\right)\cup^{\text{​}}{\left(F,\;A\right)}^{c},\;\phi \right)}$$

*is a vague entropy of CVSSs*.

**Theorem** **6.** *If*d*is the distance measure between CVSSs and satisfies*d((F, A), U)=d((F, A), ϕ),*then*:

$$M_{7}\left(F,\;A\right)=\frac{d\left(\left(F,\;A\right)\cap^{\text{​}}{\left(F,\;A\right)}^{c},\;\phi \right)}{d\left(\left(F,\;A\right)\cap^{\text{​}}{\left(F,\;A\right)}^{c},\;U\right)}$$

*is a vague entropy of CVSSs*.

**Theorem** **7.** *If*d*is a distance measure between CVSSs that satisfies*:

$$d\left(\left(F,\;A\right),\;\left(G,\;B\right)\right)=d\left({\left(F,\;A\right)}^{c},\;{\left(G,\;B\right)}^{c}\right),$$

*then*$M_{6}=M_{7}.$

**Proof.**  The proof of the Theorems 5–7 can be obtained as similar to above, so we omit here.  ☐

**Theorem** **8.** *If*d*is a distance measure between CVSSs, then*:

$$M_{8}\left(F,\;A\right)=1-d\left(\left(F,\;A\right)\cap^{\text{​}}{\left(F,\;A\right)}^{c},\;U\right)+d\left(\left(F,\;A\right)\cup^{\text{​}}{\left(F,\;A\right)}^{c},\;U\right)$$

*is a vague entropy of CVSSs*.

**Theorem** **9.** *If*d*is a distance measure between CVSSs, then*:

$$M_{9}\left(F,\;A\right)=1-d\left(\left(F,\;A\right)\cup^{\text{​}}{\left(F,\;A\right)}^{c},\;\phi \right)+d\left(\left(F,\;A\right)\cap^{\text{​}}{\left(F,\;A\right)}^{c},\;\phi \right)$$

*is a vague entropy of CVSSs*.

**Theorem** **10.** *If*d*is a distance measure between CVSSs (F, A) and (G, B) such that*:

$$d\left(\left(F,\;A\right),\;\left(G,\;B\right)\right)=d\left({\left(F,\;A\right)}^{c},\;{\left(G,\;B\right)}^{c}\right),$$

*then*$M_{8}=M_{9}.$

**Theorem** **11.** *If*d*is a distance measure between CVSSs, then*:

$$M_{10}\left(F,\;A\right)=1-d\left(\left(F,\;A\right)\cup^{\text{​}}{\left(F,\;A\right)}^{c},\;\phi \right)+d\left(\left(F,\;A\right)\cup^{\text{​}}{\left(F,\;A\right)}^{c},\;U\right)$$

*is a vague entropy of CVSSs*.

**Theorem** **12.** *If*d*is a distance measure between CVSSs, then*:

$$M_{11}\left(F,\;A\right)=1-d\left(\left(F,\;A\right)\cap^{\text{​}}{\left(F,\;A\right)}^{c},\;U\right)+d\left(\left(F,\;A\right)\cap^{\text{​}}{\left(F,\;A\right)}^{c},\;\phi \right)$$

*is a vague entropy of CVSSs*.

**Theorem** **13.** *If*d*is a distance measure between CVSSs (F,A) and (G,B) such that*:

$$d\left(\left(F,\;A\right),\;\left(G,\;B\right)\right)=d\left({\left(F,\;A\right)}^{c},\;{\left(G,\;B\right)}^{c}\right),$$

*then*$M_{10}=M_{11}.$

**Proof.**  The proof of these Theorems can be obtained as similar to above, so we omit here.  ☐

## 5. Illustrative Example

In this section, we present a scenario which necessitates the use of CVSSs. Subsequently, we present an application of the entropy measures proposed in Section 4 to an image detection problem to illustrate the validity and effectiveness of our proposed entropy formula. 

Firstly, we shall define the distance between any two CVSSs as follows:

**Definition** **10.** *Let*(F, A)*and*(G, B)*be two CVSSs over*U.*The distance between*(F, A)*and*(G, B) is as given below:

$$d\left(\left(F,\;A\right),\;\left(G,\;B\right)\right)=\frac{1}{4mn}\overset{n}{\underset{j=1}{\sum }}\overset{m}{\underset{i=1}{\sum }}[\begin{array}{c} \max\left\{\left|r_{t_{F_{\left(a_{i}\right)}}}\left(x_{j}\right)-r_{t_{G_{\left(b_{i}\right)}}}\left(x_{j}\right)\right|,\;\left|k_{f_{G_{\left(b_{i}\right)}}}\left(x_{j}\right)-k_{f_{F_{\left(a_{i}\right)}}}\left(x_{j}\right)\right|\right\}\\ +\frac{1}{2\pi }\left(\max\left\{\left|w_{t_{F_{\left(a_{i}\right)}}}^{r}\left(x_{j}\right)-w_{t_{G_{\left(b_{i}\right)}}}^{r}\left(x_{j}\right)\right|,\;\left|w_{f_{G_{\left(b_{i}\right)}}}^{k}\left(x_{j}\right)-w_{f_{F_{\left(a_{i}\right)}}}^{k}\left(x_{j}\right)\right|\right\}\right) \end{array}]$$

In order to demonstrate the utility of the above proposed entropy measures $M_{i}\left(i=1,2,\ldots ,\;11\right)$, we demonstrate it with a numerical example. For it, consider a CVSS (F,A) whose data sets are defined over the parameters $e_{1},\;e_{2}\in E$ and $x_{1},x_{2},x_{3}\in U$ as follows:

$$\left(F,A\right)=\begin{array}{c} x_{1}\\ x_{2} \end{array}[\begin{array}{ccc} \left[0.2,\;0.8\right]e^{i\left[0.1\left(2\pi \right),\;0.2\left(2\pi \right)\right]} & \left[0.3,\;0.5\right]e^{i\left[0.2\left(2\pi \right),\;0.4\left(2\pi \right)\right]} & \left[0.3,\;0.6\right]e^{i\left[0.4\left(2\pi \right),\;0.5\left(2\pi \right)\right]}\\ \left[0.3,\;0.6\right]e^{i\left[0.4\left(2\pi \right),\;0.5\left(2\pi \right)\right]} & \left[0.2,\;0.3\right]e^{i\left[0.2\left(2\pi \right),\;0.4\left(2\pi \right)\right]} & \left[0.7,\;0.9\right]e^{i\left[0.4\left(2\pi \right),\;0.5\left(2\pi \right)\right]} \end{array}]$$

and hence the complement of CVSS is:

$${\left(F,A\right)}^{c}=\begin{array}{c} x_{1}\\ x_{2} \end{array}[\begin{array}{ccc} \left[0.2,\;0.8\right]e^{i\left[0.8\left(2\pi \right),\;0.9\left(2\pi \right)\right]} & \left[0.5,\;0.7\right]e^{i\left[0.6\left(2\pi \right),\;0.8\left(2\pi \right)\right]} & \left[0.4,\;0.7\right]e^{i\left[0.5\left(2\pi \right),\;0.6\left(2\pi \right)\right]}\\ \left[0.2,\;0.5\right]e^{i\left[0.8\left(2\pi \right),\;0.9\left(2\pi \right)\right]} & \left[0.7,\;0.8\right]e^{i\left[0.6\left(2\pi \right),\;0.8\left(2\pi \right)\right]} & \left[0.1,\;0.3\right]e^{i\left[0.5\left(2\pi \right),\;0.6\left(2\pi \right)\right]} \end{array}]$$

Then, the distance measure based on the Definition 10, we get $\left(\left(F,A\right),{\left(F,A\right)}^{c}\right)=0.1708,\;d\left(\left(F,A\right)\cup^{\text{​}}{\left(F,A\right)}^{c},\;U\right)=0.2167,$ $d\left(\left(F,A\right)\cup^{\text{​}}{\left(F,A\right)}^{c},\;\left(F,A\right)\cap^{\text{​}}{\left(F,A\right)}^{c}\right)=0.1708,\;d\left(\left(F,A\right)\cap^{\text{​}}{\left(F,A\right)}^{c},\;U\right)=0.3875$, $d\left(\left(F,A\right)\cup^{\text{​}}{\left(F,A\right)}^{c},\;\phi \right)=0.3875$, $d\left(\left(F,A\right)\cap^{\text{​}}{\left(F,A\right)}^{c},\;\phi \right)=0.2167$. Therefore, the values of the entropy measures defined on the Theorem 1 to Theorem 11 are computed as:

(i) $M_{1}\left(F,\;A\right)=1-d\left(\left(F,\;A\right),\;{\left(F,\;A\right)}^{c}\right)$ = 1 − 0.1708 = 0.8292.
(ii) $M_{2}\left(F,\;A\right)=d\left(\left(F,\;A\right)\cup^{\text{​}}{\left(F,\;A\right)}^{c},\;U\right)$ = 0.2167.
(iii) $M_{3}\left(F,\;A\right)=1-d\left(\left(F,\;A\right)\cup^{\text{​}}{\left(F,\;A\right)}^{c},\;\left(F,\;A\right)\cap^{\text{​}}{\left(F,\;A\right)}^{c}\right)$ = 1 − 0.1708 = 0.8292.
(iv) $M_{4}\left(F,\;A\right)=\frac{d\left(\left(F,\;A\right)\cup^{\text{​}}{\left(F,\;A\right)}^{c},\;U\right)}{d\left(\left(F,\;A\right)\cap^{\text{​}}{\left(F,\;A\right)}^{c},\;U\right)}$ = $\frac{0.2167}{0.3875}=0.5592$.
(v) $M_{5}\left(F,\;A\right)=\frac{d\left(\left(F,\;A\right)\cap^{\text{​}}{\left(F,\;A\right)}^{c},\;\phi \right)}{d\left(\left(F,\;A\right)\cup^{\text{​}}{\left(F,\;A\right)}^{c},\;\phi \right)}=\frac{0.2167}{0.3875}=0.5592$.
(vi) $M_{6}\left(F,\;A\right)=\frac{d\left(\left(F,\;A\right)\cup^{\text{​}}{\left(F,\;A\right)}^{c},\;U\right)}{d\left(\left(F,\;A\right)\cup^{\text{​}}{\left(F,\;A\right)}^{c},\;\phi \right)}=\frac{0.2167}{0.3875}=0.5592$.
(vii) $M_{7}\left(F,\;A\right)=\frac{d\left(\left(F,\;A\right)\cap^{\text{​}}{\left(F,\;A\right)}^{c},\;\phi \right)}{d\left(\left(F,\;A\right)\cap^{\text{​}}{\left(F,\;A\right)}^{c},\;U\right)}=\frac{0.2167}{0.3875}=0.5592$.
(viii) $M_{8}\left(F,\;A\right)=1-d\left(\left(F,\;A\right)\cap^{\text{​}}{\left(F,\;A\right)}^{c},\;U\right)+d\left(\left(F,\;A\right)\cup^{\text{​}}{\left(F,\;A\right)}^{c},\;U\right)=1-0.3875+0.2167=0.5592$
(ix) $M_{9}\left(F,\;A\right)=1-d\left(\left(F,\;A\right)\cup^{\text{​}}{\left(F,\;A\right)}^{c},\;\phi \right)+d\left(\left(F,\;A\right)\cap^{\text{​}}{\left(F,\;A\right)}^{c},\;\phi \right)=1-0.3875+0.2167=0.5592$
(x) $M_{10}\left(F,\;A\right)=1-d\left(\left(F,\;A\right)\cup^{\text{​}}{\left(F,\;A\right)}^{c},\;\phi \right)+d\left(\left(F,\;A\right)\cup^{\text{​}}{\left(F,\;A\right)}^{c},\;U\right)=1-0.3875+0.2167=0.5592$
(xi) $M_{11}\left(F,\;A\right)=1-d\left(\left(F,\;A\right)\cap^{\text{​}}{\left(F,\;A\right)}^{c},\;U\right)+d\left(\left(F,\;A\right)\cap^{\text{​}}{\left(F,\;A\right)}^{c},\;\phi \right)=1-0.3875+0.2167=0.5592$

Next, we give an illustrative example from the field of pattern recognition which are stated and demonstrated as below.

### 5.1. The Scenario

**Figure 1:**
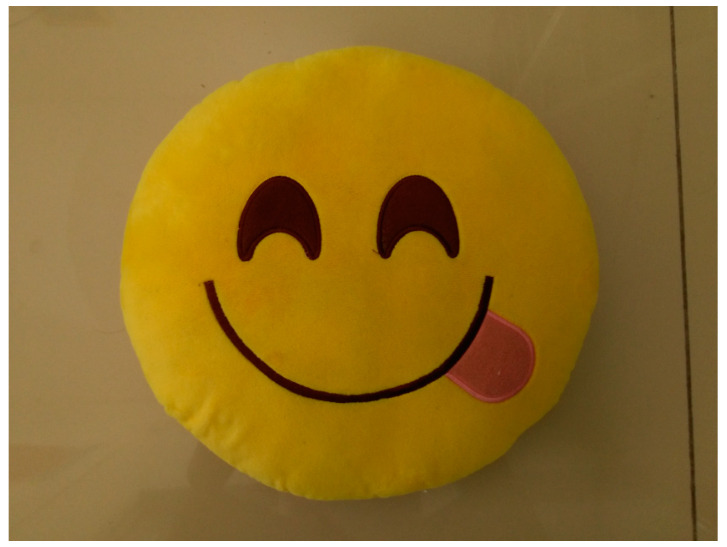
The image of the object captured by the robot.

A type of robot has a single eye capable of capturing (and hence memorizing) things it sees as an 850 × 640, 24 bit bitmap image. The robot was shown an object (a pillow with a smiley), and the image that was captured by the robot’s eye at that instant is shown in Figure 1. This image was saved as pic001.bmp in the memory of the robot. 

**Figure 2:**
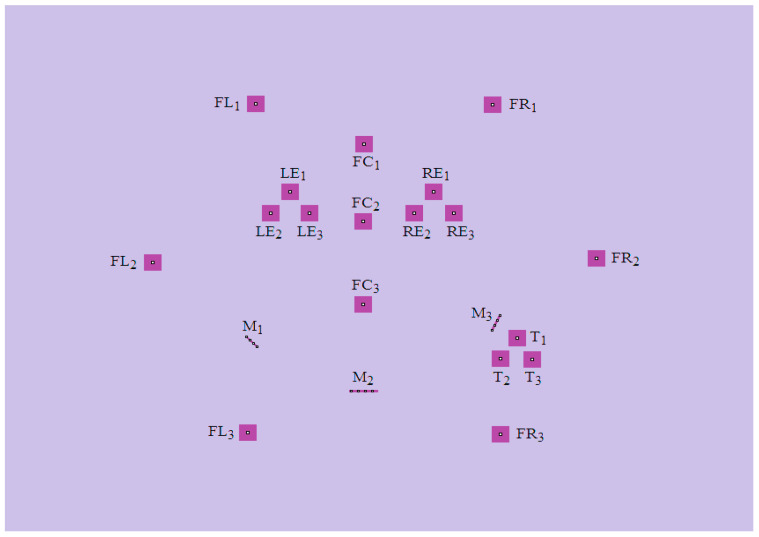
The clusters of the image pic001.bmp for recognition purposes.

The robot was then given a way (in this example, it is done by human input) to recognize the object, whenever the robot encounters the object again, by retrieving the colors at certain coordinates of its field of vision, and then comparing this with the same coordinates from image pic001.bmp stored in its memory. In order to distinguish noises, the coordinates are chosen in clusters of four, as shown in Figure 2. The coordinates of the clusters of the images are summarized in Table 1.

**Figure 3:**
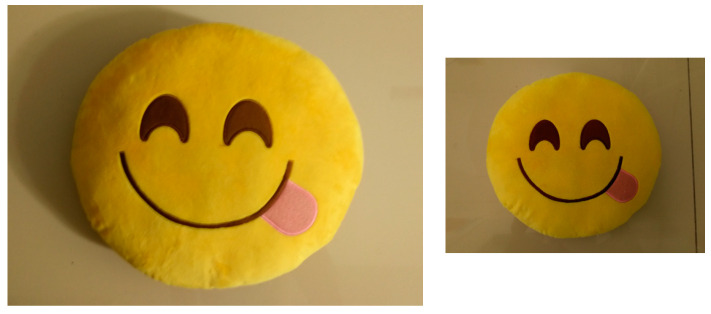
Image A (**left**) and the original image pic001.bmp (**right**).

**Figure 4:**
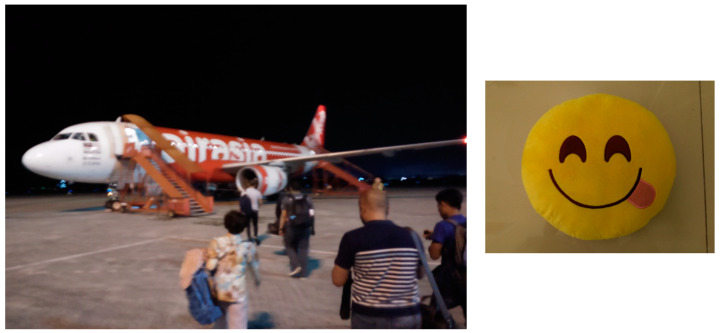
Image B (**left**) and the original image pic001.bmp (**right**).

**Figure 5:**
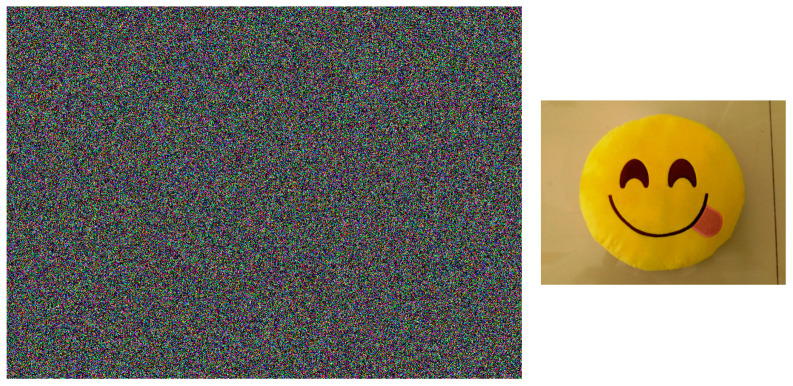
Image C (**left**) and the original image pic001.bmp (**right**).

We now have three images, namely image A, image B and image C. The robot needs to recognize if the objects shown in image A, B and C is the same as the image shown in image pic001.bmp stored in the robot’s memory. Images A, B and C are shown in Figure 3, Figure 4 and Figure 5, respectively. For comparison purposes, pic001.bmp is shown alongside all the three images.

From a human perspective, it is clear that the object shown in image A will be recognized as the same object shown in image pic001.bmp, and it will be concluded that the object is shown in image B (a red airplane) is not the image pic001.bmp stored in the memory of the robot. No conclusion can be deduced from image C as it is made up of only noise, and therefore we are unable to deduce the exact object behind the noise. By retrieving the coordinates from Table 1, we now obtain the following sets of colors which are given in Table 2. 

The luminosity and hue of the pixels are obtained using a picture editing program, and these are given in Table 3 and Table 4, respectively. 

Luminosity, $L_{m,k,n,b}$, where k ∈ {LE, RE, LF, CF, RF, T, M}, *b* ∈ {0, 1, 2, 3} (cluster of four pixels). Hue, ${ℌ}_{m,k,n,b}$, where k ∈ {LE, RE, LF, CF, RF, T, M}, *b* ∈ {0, 1, 2, 3} (cluster of four pixels).

### 5.2. Formation of CVSS and Calculation of Entropies

Let $U=\left\{x_{1},x_{2},x_{3}\right\},$ and A= {LE, RE, LF, CF, RF, T, M}. We now form three CVSSs $\left({ℱ}_{𝒷},\;A\right),𝒷\in \left\{1,2,3\right\},$ which denote image A, B and C, respectively using the formula given below:

$${ℱ}_{{𝒷}_{\left(k\right)}}\left(x_{n}\right)=\left[e^{-\left(\frac{{\left(y_{𝒷,\left(k,n\right)}\right)}^{2}}{\rho }\right)},e^{-\left(\frac{{\left(\gamma_{𝒷,\left(k,n\right)}\right)}^{2}}{\rho }\right)}\right]e^{2\pi i\left[e^{-\left(\frac{{\left(\theta_{𝒷,\left(k,n\right)}\right)}^{2}}{\varrho }\right)},e^{-\left(\frac{{\left(\vartheta_{𝒷,\left(k,n\right)}\right)}^{2}}{\varrho }\right)}\right]},$$

where:

$$y_{𝒷,\left(k,n\right)}=\min\left\{\left|L_{𝒷,k,n,p}-L_{0,k,n,q}\right|\;:\;p,q\in \left\{0,1,2,3\right\}\right\},\gamma_{𝒷,\left(k,n\right)}=\max\left\{\left|L_{𝒷,k,n,p}-L_{0,k,n,q}\right|\;:\;p,q\in \left\{0,1,2,3\right\}\right\},$$

$$\theta_{𝒷,\left(k,n\right)}=\min\left\{\left|{ℌ}_{𝒷,k,n,p}-{ℌ}_{0,k,n,q}\right|\;:\;p,q\in \left\{0,1,2,3\right\}\right\},\vartheta_{𝒷,\left(k,n\right)}=\max\left\{\left|{ℌ}_{𝒷,k,n,p}-{ℌ}_{0,k,n,q}\right|\;:\;p,q\in \left\{0,1,2,3\right\}\right\}.$$

We choose ρ=6400 and ϱ=6400 for this scenario. The CVSSs that were formed for this scenario are as given in Table 5, Table 6 and Table 7.

By using Definition 10, the entropy values for images A, B and C are as summarized in Table 8. 

From these values, it can be clearly seen that $M_{i}\left({ℱ}_{3},\;A\right)>M_{i}\left({ℱ}_{2},\;A\right)>M_{i}\left({ℱ}_{1},\;A\right)$ for all i=1, 2, …, 11. Hence it can be concluded that Image A is the image that is closest to the original image pic001.bmp that is stored in the memory of the robot, whereas Image C is the image that is the least similar to the original pic001.bmp image. The high entropy value for $\left({ℱ}_{3},\;A\right)$ is also an indication of the abnormality of Image C compared to Images A and B. These entropy values and the results obtained for this scenario prove the effectiveness of our proposed entropy formula. The entropy values obtained in Table 8 further verifies the validity of the relationships between the 11 formulas that was proposed in Section 4.

## 6. Conclusions

The objective of this work is to introduce some entropy measures for the complex vague soft set environment to measure the degree of the vagueness between sets. For this, we define firstly the axiomatic definition of the distance and entropy measures for two CVSSs and them some desirable relations between the distance and entropy are proposed. The advantages of the proposed measures are that they are defined over the set where the membership and non-membership degrees are defined as a complex number rather than real numbers. All of the information measures proposed here complement the CVSS model in representing and modeling time-periodic phenomena. The proposed measures are illustrated with a numerical example related to the problem of image detection by a robot. Furthermore, the use of CVSSs enables efficient modeling of the periodicity and/or the non-physical attributes in signal processing, image detection, and multi-dimensional pattern recognition, all of which contain multi-dimensional data. The work presented in this paper can be used as a foundation to further extend the study of the information measures for complex fuzzy sets or its generalizations. On our part, we are currently working on studying the inclusion measures and developing clustering algorithms for CVSSs. In the future, the result of this paper can be extended to some other uncertain and fuzzy environment [59,60,61,62,63,64,65,66,67,68].

## Figures and Tables

**Table 1:** The coordinates of the clusters for image pic001.bmp from Figure 2.

	1st Position (*n* = 1)	2nd Position (*n* = 2)	3rd Position (*n* = 3)
“Left Eye” (LE*_n_*)	(323, 226), (324, 226), (323, 227), (324, 227),	(301, 252), (302, 252), (301, 253), (302, 253),	(345, 252), (346, 252), (345, 253), (346, 253),
“Right Eye” (RE*_n_*)	(486, 226), (487, 226), (486, 227), (487, 227),	(464, 252), (465, 252), (464, 253), (465, 253),	(509, 252), (510, 252), (509, 253), (510, 253),
“Left side of Face” (LF*_n_*)	(284, 119), (285, 119), (284, 120), (285, 120),	(167, 312), (168, 312), (167, 313), (168, 313),	(275, 519), (276, 519), (275, 520), (276, 520),
“Centre of Face” (CF*_n_*)	(407, 168), (408, 168), (407, 169), (408, 169),	(406, 262), (407, 262), (406, 263), (407, 263),	(406, 363), (407, 363), (406, 364), (407, 364),
“Right side of Face” (RF*_n_*)	(553, 120), (554, 120), (553, 121), (554, 121),	(671, 307), (672, 307), (671, 308), (672, 308),	(562, 521), (563, 521), (562, 522), (563, 522),
“Tongue” (T*_n_*)	(581, 404), (582, 404), (581, 405), (582, 405),	(562, 429), (563, 429), (562, 430), (563, 430),	(598, 430), (599, 430), (598, 431), (599, 431),
“Mouth” (M*_n_*)	(274, 403), (278, 407), (282, 411), (286, 415),	(393, 469), (401, 469), (409, 469), (417, 469),	(553, 395), (556, 389), (559, 383), (562, 377),

Remark: For a computer image, the top-leftmost pixel is labeled (0, 0).

**Table 2:** The sets of colors for image A, B, C and image pic001.bmp.

	pic001.bmp (Memory)			Image A			Image B			Image C		
	*n* = 1	*n* = 2	*n* = 3	*n* = 1	*n* = 2	*n* = 3	*n* = 1	*n* = 2	*n* = 3	*n* = 1	*n* = 2	*n* = 3
LE	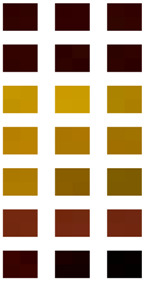			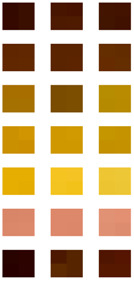			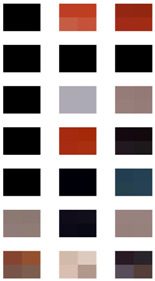			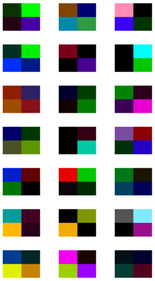		
RE												
LF												
CF												
RF												
T												
M												

**Table 3:** The values of the luminosity for image A, B, C and image pic001.bmp.

	pic001.bmp (Memory), *m* = 0						Image A *m* = 1						Image B *m* = 2						Image C *m* = 3					
	*n* = 1		*n* = 2		*n* = 3		*n* = 1		*n* = 2		*n* = 3		*n* = 1		*n* = 2		*n* = 3		*n* = 1		*n* = 2		*n* = 3	
LE	23	23	23	24	24	23	24	27	34	33	32	32	5	6	97	96	74	72	27	128	57	54	180	2
	24	24	24	24	24	25	25	28	38	39	31	31	6	6	116	110	81	80	25	82	78	107	120	26
RE	24	23	21	24	23	24	43	44	41	42	47	46	7	7	8	7	8	7	28	112	48	0	2	120
	23	24	22	21	24	22	45	46	41	43	48	48	6	6	8	8	7	7	120	64	0	67	0	97
LF	101	99	104	105	90	90	78	79	55	57	96	96	3	3	162	163	122	120	58	63	24	31	62	24
	96	100	106	106	91	88	78	78	55	56	95	95	3	3	163	163	125	122	65	67	14	61	40	96
CF	85	88	80	83	78	79	97	102	103	104	102	101	6	5	79	77	24	24	49	28	0	22	109	56
	88	89	81	82	78	78	97	98	103	104	99	100	5	5	81	81	33	32	60	81	3	139	27	96
RF	83	82	64	64	60	59	119	119	136	136	139	141	6	6	16	15	62	63	98	40	93	94	56	16
	84	84	65	63	59	59	120	119	135	134	138	139	6	6	16	14	61	64	64	0	20	30	45	44
T	60	60	59	59	58	59	142	143	144	144	152	151	116	117	27	27	127	127	74	28	0	75	81	198
	59	61	61	62	60	59	144	144	145	144	150	148	120	119	25	24	125	125	120	25	104	3	0	75
M	26	23	15	14	9	9	19	21	34	40	33	35	81	87	175	191	36	42	77	22	112	15	14	24
	25	26	13	13	8	8	19	20	43	40	36	36	86	93	181	145	79	66	113	84	121	115	31	33

**Table 4:** The values of the hue of the pixels for image A, B, C and image pic001.bmp.

	pic001.bmp (Memory), *m* = 0						Image A *m* = 1						Image B *m* = 2						Image C *m* = 3					
	*n* = 1		*n* = 2		*n* = 3		*n* = 1		*n* = 2		*n* = 3		*n* = 1		*n* = 2		*n* = 3		*n* = 1		*n* = 2		*n* = 3	
LE	12	12	10	10	12	12	17	17	15	15	18	16	187	160	8	8	6	7	55	80	23	160	226	27
	13	12	9	10	13	12	17	17	15	15	18	16	160	160	8	8	6	7	214	173	127	70	168	66
RE	13	13	13	13	12	11	19	19	18	18	20	20	160	160	160	160	160	160	112	64	227	160	220	119
	13	13	13	12	12	11	19	19	18	18	19	20	160	160	160	160	160	160	152	150	160	177	160	77
LF	31	31	31	31	31	30	28	28	27	27	31	31	187	187	173	167	7	7	12	167	169	67	57	211
	31	30	31	31	31	30	29	28	27	27	31	30	187	187	171	167	9	7	23	237	18	76	186	199
CF	29	29	29	28	29	29	30	29	30	30	31	30	160	160	8	8	224	230	155	86	160	214	177	0
	29	29	29	29	29	29	30	29	30	30	30	30	180	180	8	8	213	220	42	51	200	107	97	165
RF	29	30	29	29	31	31	30	30	32	32	33	33	160	160	160	160	139	139	154	5	238	68	94	36
	30	30	29	29	31	31	30	30	32	32	32	33	160	160	160	153	137	141	68	160	192	76	131	158
T	11	11	11	12	10	10	12	12	12	12	13	12	13	9	168	165	7	7	119	212	160	45	160	130
	11	11	9	9	9	10	12	12	11	12	13	12	9	13	164	160	8	10	29	205	31	67	160	200
M	11	10	13	13	18	14	16	15	18	20	17	16	10	14	19	18	208	183	145	118	195	26	124	160
	11	12	13	13	14	18	15	16	21	19	16	17	13	14	17	15	184	5	42	29	49	181	115	220

**Table 5:** Tabular representation of $\left({ℱ}_{1},\;A\right)$.

		*n*		
		1	2	3
*k*	LE	[0.996, 1.000]*e*^2*πi*[0.996, 0.997]^	[0.960, 0.987]*e*^2*πi*[0.994, 0.996]^	[0.987, 0.994]*e*^2*πi*[0.994, 0.998]^
	RE	[0.920, 0.945]*e*^2*πi*[0.994, 0.994]^	[0.927, 0.955]*e*^2*πi*[0.994, 0.996]^	[0.899, 0.927]*e*^2*πi*[0.987, 0.992]^
	LF	[0.920, 0.955]*e*^2*πi*[0.998, 0.999]^	[0.666, 0.708]*e*^2*πi*[0.997, 0.997]^	[0.990, 0.997]*e*^2*πi*[0.999, 1.000]^
	CF	[0.955, 0.990]*e*^2*πi*[0.999, 1.000]^	[0.913, 0.939]*e*^2*πi*[0.999, 0.999]^	[0.913, 0.939]*e*^2*πi*[0.999, 0.999]^
	RF	[0.798, 0.825]*e*^2*πi*[0.999, 1.000]^	[0.434, 0.475]*e*^2*πi*[0.998, 0.998]^	[0.349, 0.386]*e*^2*πi*[0.999, 0.999]^
	T	[0.323, 0.358]*e*^2*πi*[0.999, 0.999]^	[0.314, 0.349]*e*^2*πi*[0.998, 1.000]^	[0.251, 0.298]*e*^2*πi*[0.997, 0.999]^
	M	[0.992, 0.999]*e*^2*πi*[0.994, 0.998]^	[0.868, 0.945]*e*^2*πi*[0.990, 0.996]^	[0.884, 0.913]*e*^2*πi*[0.998, 0.999]^

**Table 6:** Tabular representation of $\left({ℱ}_{2},\;A\right)$.

		*N*		
		1	2	3
*k*	LE	[0.945, 0.955]*e*^2*πi*[0.008, 0.034]^	[0.258, 0.444]*e*^2*πi*[0.999, 0.999]^	[0.591, 0.708]*e*^2*πi*[0.992, 0.996]^
	RE	[0.950, 0.960]*e*^2*πi*[0.034, 0.034]^	[0.955, 0.973]*e*^2*πi*[0.032, 0.034]^	[0.955, 0.969]*e*^2*πi*[0.031, 0.032]^
	LF	[0.222, 0.258]*e*^2*πi*[0.021, 0.022]^	[0.580, 0.612]*e*^2*πi*[0.042, 0.055]^	[0.807, 0.876]*e*^2*πi*[0.913, 0.933]^
	CF	[0.332, 0.377]*e*^2*πi*[0.028, 0.068]^	[0.994, 1.000]*e*^2*πi*[0.933, 0.939]^	[0.623, 0.728]*e*^2*πi*[0.001, 0.005]^
	RF	[0.386, 0.405]*e*^2*πi*[0.068, 0.071]^	[0.666, 0.708]*e*^2*πi*[0.068, 0.090]^	[0.996, 0.999]*e*^2*πi*[0.150, 0.172]^
	T	[0.559, 0.623]*e*^2*πi*[0.999, 0.999]^	[0.798, 0.852]*e*^2*πi*[0.019, 0.032]^	[0.475, 0.516]*e*^2*πi*[0.998, 1.000]^
	M	[0.465, 0.623]*e*^2*πi*[0.997, 1.000]^	[0.007, 0.071]*e*^2*πi*[0.994, 0.999]^	[0.454, 0.892]*e*^2*πi*[0.002, 0.987]^

**Table 7:** Tabular representation of $\left({ℱ}_{3},\;A\right)$.

		*N*		
		1	2	3
*k*	LE	[0.178, 0.999]*e*^2*πi*[0.001, 0.759]^	[0.332, 0.868]*e*^2*πi*[0.028, 0.973]^	[0.021, 0.999]*e*^2*πi*[0.000, 0.969]^
	RE	[0.229, 0.997]*e*^2*πi*[0.048, 0.666]^	[0.718, 0.933]*e*^2*πi*[0.000, 0.034]^	[0.222, 0.939]*e*^2*πi*[0.001, 0.516]^
	LF	[0.749, 0.876]*e*^2*π*i[0.001, 0.992]^	[0.266, 0.749]*e*^2*πi*[0.051, 0.973]^	[0.495, 0.996]*e*^2*πi*[0.005, 0.899]^
	CF	[0.559, 0.997]*e*^2*πi*[0.083, 0.973]^	[0.340, 0.612]*e*^2*πi*[0.004, 0.386]^	[0.655, 0.955]*e*^2*πi*[0.032, 0.876]^
	RF	[0.332, 0.969]*e*^2*πi*[0.068, 0.913]^	[0.728, 0.884]*e*^2*πi*[0.001, 0.788]^	[0.738, 0.998]*e*^2*πi*[0.080, 0.996]^
	T	[0.559, 0.973]*e*^2*πi*[0.001, 0.950]^	[0.548, 0.973]*e*^2*πi*[0.028, 0.945]^	[0.046, 0.965]*e*^2*πi*[0.003, 0.105]^
	M	[0.282, 0.999]*e*^2*πi*[0.057, 0.955]^	[0.161, 1.000]*e*^2*πi*[0.005, 0.973]^	[0.906, 0.996]*e*^2*πi*[0.001, 0.229]^

**Table 8:** Summary of the entropy values for image A, B and C.

Entropy Measure	Image A $\left({ℱ}_{1},\;A\right)$	Image B $\left({ℱ}_{2},\;A\right)$	Image C $\left({ℱ}_{3},\;A\right)$
$M_{1}\left({ℱ}_{i},\;A\right)$	0.571	0.647	0.847
$M_{2}\left({ℱ}_{i},\;A\right)$	0.039	0.089	0.328
$M_{3}\left({ℱ}_{i},\;A\right)$	0.571	0.647	0.847
$M_{4}\left({ℱ}_{i},\;A\right)$	0.084	0.202	0.682
$M_{5}\left({ℱ}_{i},\;A\right)$	0.084	0.202	0.682
$M_{6}\left({ℱ}_{i},\;A\right)$	0.084	0.202	0.682
$M_{7}\left({ℱ}_{i},\;A\right)$	0.084	0.202	0.682
$M_{8}\left({ℱ}_{i},\;A\right)$	0.571	0.647	0.847
$M_{9}\left({ℱ}_{i},\;A\right)$	0.571	0.647	0.847
$M_{10}\left({ℱ}_{i},\;A\right)$	0.571	0.647	0.847
$M_{11}\left({ℱ}_{i},\;A\right)$	0.571	0.647	0.847
